# Debonding of the orthodontic brackets; is diode laser a safer method? Experimental invitro study

**DOI:** 10.1186/s12903-025-07516-8

**Published:** 2026-01-15

**Authors:** Eman M. Salem, S. M. Khattab

**Affiliations:** 1 Oral Biology Department, Pharos University (PUA), P.O. Box 37, Sidi Gaber, Alexandria, Egypt; 2 Cairo University Hospital, Cairo University, Cairo, Egypt; 3 Department of Orthodontics, Pharos University, Alexandria, Egypt

**Keywords:** Debonding, Diode laser, Orthodontic brackets, Shear bond strength, Enamel surface, Scanning electron microscopy.

## Abstract

**Abstract:**

Bracket debonding remains a clinical challenge in orthodontics, particularly when removing ceramic brackets, due to the potential risk of enamel cracks and surface damage. Recently, laser-assisted techniques have been investigated to facilitate safer and less traumatic bracket removal. This study aimed to evaluate the effect of 940 nm diode laser irradiation on the debonding process of metallic and ceramic orthodontic brackets by assessing shear bond strength (SBS) and enamel surface morphology using scanning electron microscopy (SEM).

**Methods:**

Thirty two extracted human premolars were randomly divided into four experimental groups: Group A (Control–Metallic),Group B (Control–Ceramic),Group C (Laser–Metallic), and Group D (Laser–Ceramic).Brackets were bonded using a light-cured adhesive and subjected to diode laser irradiation at 940 nm, 1 W, for 5 seconds in the laser-treated groups. SBS was measured using a universal testing machine at a crosshead speed of 1 mm/min, and mean values were expressed in MPa. Enamel surface morphology was analyzed using SEM at 80× and 1000× magnifications. Statistical analysis was performed using one-way ANOVA and independent t-tests with a significance level of p < 0.05.

**Results:**

Laser-assisted debonding significantly reduced SBS in metallic brackets (mean ± SD: 2.4 ± 0.3 MPa) compared with the control group (4.3 ± 0.4 MPa) (p < 0.05). In contrast, the difference between laser-treated and control ceramic brackets was not statistically significant. SEM observations revealed smoother enamel surfaces and fewer cracks in the laser-treated groups compared with controls.

**Conclusions:**

Diode laser–assisted debonding (940 nm, 1 W) effectively decreases shear bond strength for metallic brackets and minimizes enamel surface alterations, suggesting it is a promising adjunct for safer bracket removal. However, the technique’s effect on ceramic brackets appears limited at this power setting.

## Introduction

Orthodontic treatment aims to achieve ideal occlusion and facial harmony; however, the final step—debonding of brackets—remains a critical and sometimes hazardous procedure. Excessive force during bracket removal can lead to enamel fractures, cracks, or surface loss, compromising the esthetic outcome and patient satisfaction [1]. The risk of enamel damage is particularly high when ceramic brackets are used, due to their high brittleness and low fracture toughness, which cause stress concentration at the adhesive–enamel interface during mechanical debonding [2].

Conventional mechanical removal methods rely on pliers or torsional forces to detach the bracket, often generating uncontrolled shear stresses that propagate through the enamel prisms. These stresses can cause irreversible enamel cracks, residual adhesive remnants, or even bracket fracture [3, 4]. To minimize these complications, laser-assisted debonding techniques have been proposed as a more conservative and patient-friendly alternative [5].

Different laser systems, such as CO₂, Er: YAG, Nd: YAG, and diode lasers, have been investigated for orthodontic bracket debonding [6]. The principle is based on localized heating of the adhesive resin, leading to its softening and subsequent reduction in shear bond strength (SBS). The laser type and wavelength determine how energy is absorbed and distributed across the bracket–adhesive–enamel complex [3].

Among these systems, diode lasers, typically operating between 810 nm and 940 nm, are especially advantageous because of their compact design, affordability, and selective absorption by pigmented adhesive components [3]. When used appropriately, diode laser energy can soften the resin without causing excessive intrapulpal temperature rise, maintaining thermal safety thresholds (< 5.5 °C) [4].

Laser–enamel interactions also depend on bracket material. Metallic brackets reflect a considerable portion of laser energy, reducing heat transmission to the adhesive, whereas ceramic brackets, being translucent, allow deeper light penetration and greater thermal diffusion [7]. Consequently, the optimal laser parameters differ for metallic and ceramic brackets to achieve efficient debonding without damaging enamel tissue.

Recent studies have shown that diode laser irradiation can significantly reduce SBS values for both bracket types while maintaining enamel integrity and pulpal safety [8]. However, reported outcomes remain inconsistent due to variations in power output, exposure time, and study design [8]. A 2022 *BMC Oral Health* study by Khalil et al. demonstrated the potential of diode laser-assisted debonding but highlighted the need for further research focusing on microstructural enamel changes rather than bond strength alone [9].

Therefore, this study aimed to evaluate the effect of 940 nm diode laser irradiation on the debonding of metallic and ceramic orthodontic brackets, analyzing both quantitative (SBS) and qualitative (SEM) outcomes. The novelty of this investigation lies in its focus on the histologic and microstructural preservation of enamel, providing insight into whether diode laser use can offer a safer and more biologically conservative method for bracket removal compared with conventional techniques.

## Materials and methods

### Study design and ethical approval

This was an in-vitro experimental study designed to evaluate the effect of 940 nm diode-laser irradiation on the debonding of metallic and ceramic orthodontic brackets, assessing both shear bond strength (SBS) and enamel surface morphology.

The protocol was approved by the Institutional Review Board of the Faculty of Dentistry, [Pharos University, Alexandria, Egypt under ethical code 393.]. The study followed the ethical standards of the Helsinki Declaration for research using human tissues.

### Tooth selection and storage

Forty five (45) freshly extracted human premolars were collected from orthodontic patients aged 15–25 years who required extractions for therapeutic reasons. Inclusion criteria were: intact buccal enamel, no caries, cracks, restorations, or fluorosis. Thus, the sample size after exclusion was thirty-two (32) teeth, Immediately after extraction, the teeth were cleaned of soft-tissue remnants and stored in 0.1% thymol solution at 4 °C to prevent microbial growth and preserve mineral content until testing [10].

Before bonding, the teeth were rinsed thoroughly with distilled water and mounted vertically in self-curing acrylic resin blocks so that only the crown was exposed.

### Grouping of samples

Specimens were randomly allocated into four experimental groups (*n* = 8 each) according to bracket type and debonding technique (Table 1):

A Metallic bracket Conventional mechanical debonding (control)
B Ceramic bracket Conventional mechanical debonding (control)
C Metallic bracket Diode-laser–assisted debonding
D Ceramic bracket Diode-laser–assisted debonding

**Table 1:** Showing the results of SBS measuring for control groups

**Metal Control**	**Force**	**SBS**	**Ceramic Control**	**Force**	**SBS**
1	99.5	7.4	1	37.8	2.8
2	66.7	4.9	2	35.1	2.6
3	24.5	1.8	3	78.6	5.8
4	63.0	4.7	4	82.6	6.1
5	47.9	3.5	5	49.0	3.6
6	56.6	4.2	6	89.8	6.7
7	55.1	4.1	7	83.9	6.2
8	52.3	3.9	8	48.5	3.6
Average		4.3	Average		4.7

### Bonding procedure

The buccal enamel surface of each specimen was polished with pumice slurry using a rubber cup for 10 s, rinsed, and air-dried. The surface was then etched with 37% phosphoric-acid gel for 30 s, rinsed with water for 20 s, and air-dried until a frosty white appearance was visible.

**Fig. 1:**
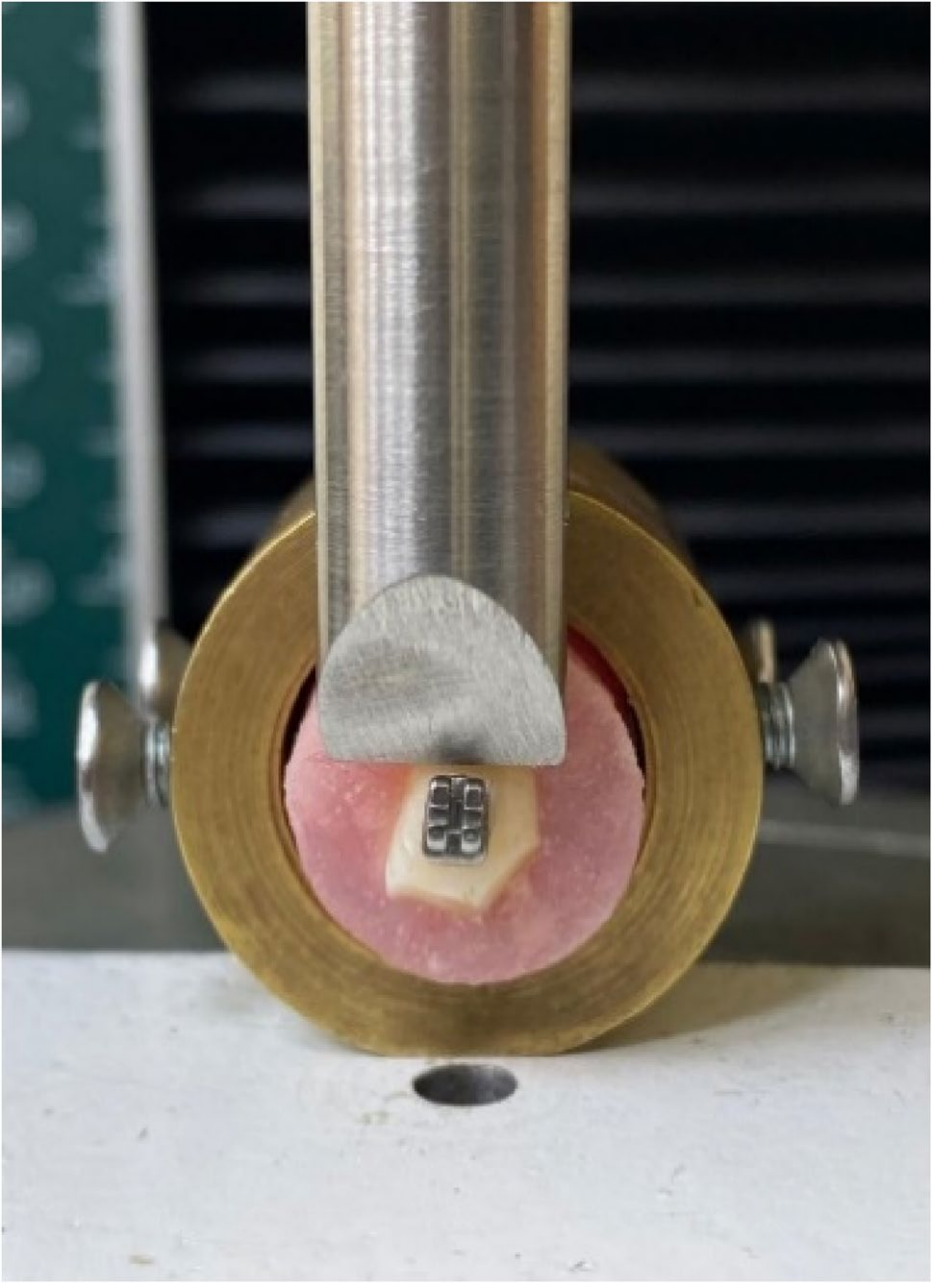
Measuring the SBS for the metallic bracket

A thin layer of Transbond XT primer (3 M Unitek, USA) was applied followed by Transbond XT adhesive paste. Stainless-steel or ceramic premolar edgewise brackets (0.022-inch slot, American Orthodontics, USA) were positioned and pressed firmly in place; excess resin was removed (Fig. 1). 

Polymerization was performed using an LED curing unit (Elipar S10, 3 M ESPE, USA) for 20 s total (10 s mesial + 10 s distal). All specimens were stored in distilled water at 37 °C for 24 h to complete resin polymerization [11].

### Laser irradiation protocol

Groups C and D were irradiated with a 940 nm diode laser (Epic X, Biolase Inc., USA) in continuous-wave mode. The laser was delivered through a 300 μm fiber-optic tip positioned 2 mm from the bracket surface, moved in a sweeping horizontal motion across the bracket base.

Operating parameters were: 1 W output power, 5 s exposure time, and spot size ≈ 0.3 mm².

Energy was applied evenly across the bracket to heat the adhesive layer by thermal conduction while avoiding enamel overheating. The selected settings were based on previously validated intrapulpal safety thresholds (< 5.5 °C temperature rise) [12].

### Shear bond strength (SBS) testing

After irradiation, each specimen was fixed in a universal testing machine (Instron 3345, USA). A chisel-shaped metal blade was applied parallel to the enamel surface at the bracket–adhesive interface, and load was applied at a crosshead speed of 0.5 mm min⁻¹ until bracket failure occurred.

The debonding force (N) was recorded and converted to SBS (MPa) using:

$$SBS = \frac{Force \left(N\right)}{Bracket\:base\:area \left(mm^{2}\right)}$$

where the bracket-base area = 10.61 mm².

### Scanning electron microscopy (SEM) analysis

Two representative teeth from each group were ultrasonically cleaned, dehydrated in ascending ethanol concentrations (70%, 80%, 90%, 100%), sputter-coated with gold, and examined using a scanning electron microscope (JEOL JSM-6510LV, Japan) at ×80, ×500, and ×1000 magnifications.

Images were analyzed for enamel micro-morphology, including cracks, prism patterns, resin remnants, and surface smoothness [13].

### Statistical analysis

Data analysis was performed using IBM SPSS Statistics, Version 24.0. The Shapiro–Wilk test confirmed the normal distribution of SBS data (*p* > 0.05). Quantitative data were presented as means and standard deviations. Independent sample t-tests were used to compare two groups, while one-way ANOVA followed by post hoc testing was applied for comparisons across more than two groups. A significance level of *p* < 0.05 was used throughout.

**Fig. 2:**
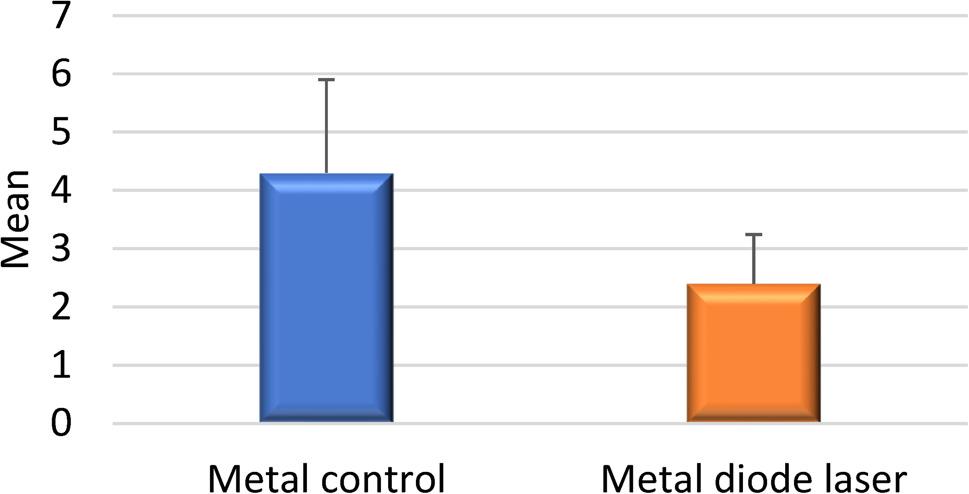
Comparison between SBS in metal control and metal diode laser

**Fig. 3:**
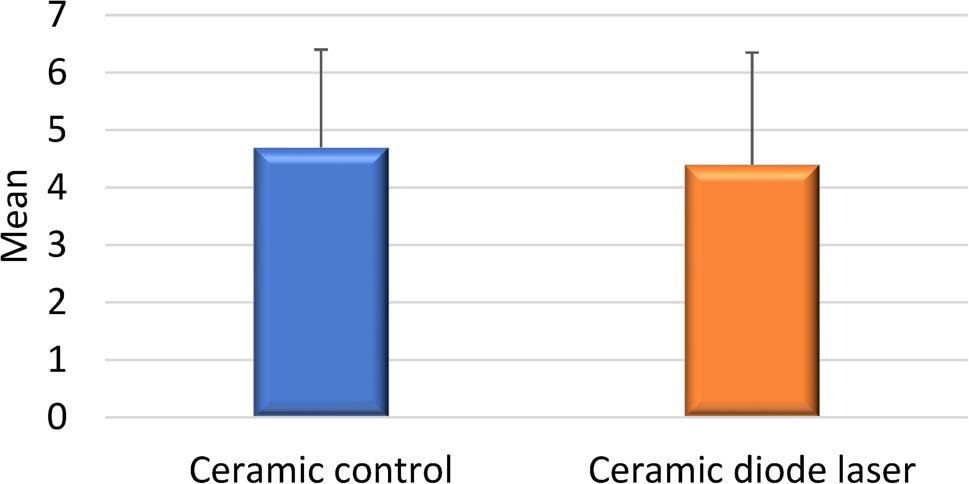
Comparison between SBS in Ceramic control and Ceramic diode laser

Analysis revealed a statistically significant difference in SBS between the metallic control group and the laser-assisted metallic group. However, no significant difference was observed between the ceramic control and laser-treated ceramic groups (Figs. 2 and 3). 

Wilk test confirmed data normality. One-way ANOVA followed by Tukey’s post-hoc test compared mean SBS among groups, while Kruskal–Wallis and. Significance was set at *p* < 0.05.

## Results

### Shear bond strength (SBS) results

Shear bond strength testing was conducted using a universal testing machine (Tinius Olsen, Model 5ST, England, 2018). For each test, the buccal surface of the tooth was aligned parallel to the applied force vector, and the bracket slot was positioned horizontally. A chisel-edge blade was placed at the bracket–enamel interface in shear mode until bond failure occurred. The debonding force was recorded in Newtons and converted into megapascals (MPa) by dividing the applied force by the surface area of the bracket base.

The mean SBS values for the four experimental groups were as follows:

- Group A (Control – Metal): 4.3 MPa.
- Group B (Control – Ceramic): 4.7 MPa.
- Group C (Laser – Metal): 2.4 MPa.
- Group D (Laser – Ceramic): 4.4 MPa.

A reduction of approximately 45% in SBS was observed in the laser-treated metallic bracket group (Group C) compared to its control (Group A), indicating a significant weakening effect of the diode laser on the adhesive bond. These values are presented in Tables 1 and 2.

**Table 2:** Showing the results of SBS measuring for lased groups

**Metal ** **Diode** **Laser**	**Force**	**SBS**	**Ceramic ** **Diode** **Laser**	**Force**	**SBS**
1	18.1	1.3	1	28.6	2.1
2	34.2	2.5	2	75.1	5.6
3	18.1	1.3	3	76.9	5.7
4	48.5	3.6	4	21.3	1.6
5	29.9	2.2	5	97.9	7.3
6	33.1	2.5	6	67.5	5.0
7	35.4	2.6	7	69.1	5.1
8	46.9	3.5	8	43.1	3.2
Average		2.4	Average		4.4

### Scanning electron microscopy (SEM) results

**Fig. 4:**
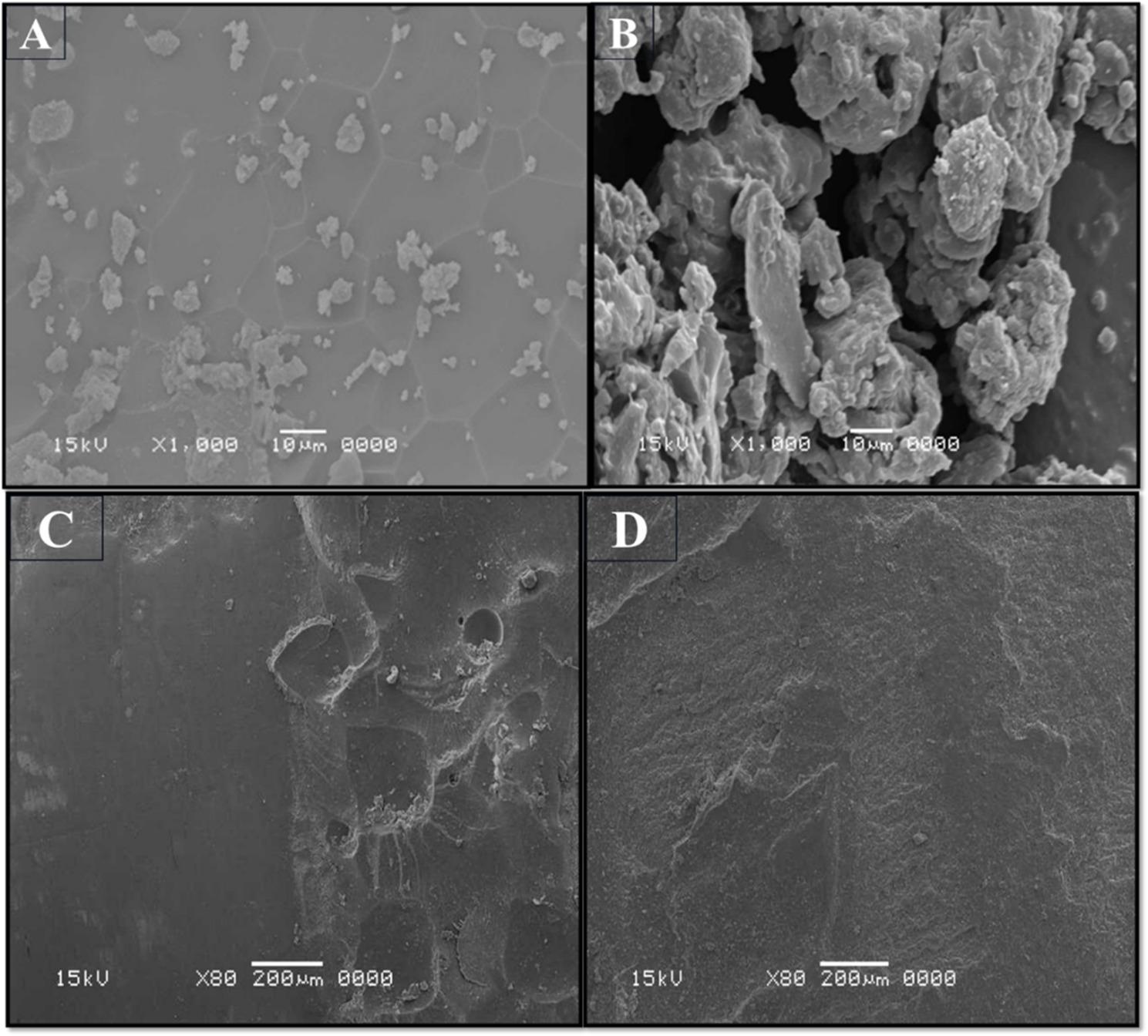
Scanning electron micrographs of enamel surfaces after bracket debonding. Groups **A** and **B** (controls) exhibit irregular enamel topography with surface projections and areas of plaque accumulation (×1000). Group **C** (metallic–laser) shows a smoother, more homogeneous surface with generalized flattening and a marked reduction in surface irregularities compared with the control (×80). Group **D** (ceramic–laser) also demonstrates a smooth, uniform enamel surface with fewer surface irregularities (×80)

Scanning electron microscopy (SEM) was employed to examine enamel surface morphology and potential damage at the microstructural level. All samples were sputter-coated with gold prior to imaging to improve conductivity and image clarity. SEM images were captured at two magnifications:80× magnification to assess the overall morphology of the debonded surface, and 1000× magnification at three specific locations: the enamel surface, the bonded region, and the bracket–enamel interface.SEM analysis revealed a higher incidence of enamel cracks and surface irregularities in the control groups (Groups A and B) compared to the laser-assisted groups (Groups C and D). The laser-assisted debonding process appeared to preserve enamel integrity more effectively displayed markedly smoother surfaces with fewer cracks and minimal adhesive remnants. Representative SEM images are shown in (Fig. 4). 

## Discussion

The present study aimed to assess the influence of 940 nm diode laser irradiation on the debonding efficiency of metallic and ceramic orthodontic brackets. The results revealed a statistically significant reduction in shear bond strength (SBS) following laser exposure in metallic brackets, whereas the effect was less pronounced in ceramic brackets. These findings indicate that diode laser–assisted debonding can facilitate safer removal of metallic brackets by minimizing the mechanical stress transmitted to the enamel surface.

### Comparison with previous studies

The current findings are in agreement with previous investigations demonstrating that diode laser irradiation effectively reduces the adhesive bond strength between orthodontic brackets and enamel [14, 15]. Comparable results were reported in studies where laser exposure at controlled power levels decreased SBS while maintaining pulpal safety. Furthermore, smoother enamel surfaces were observed following laser-assisted debonding compared with conventional mechanical techniques [16].

Nevertheless, the extent of SBS reduction appears to depend strongly on laser parameters, including wavelength, output power, and exposure duration. Investigations employing higher power levels (2.5–5 W) achieved greater reductions in SBS but also noted an increased risk of intrapulpal temperature rise [14]. In the present study, the use of a 940 nm diode laser at 1 W for 5 s achieved an optimal balance between effective debonding and enamel protection.

### Mechanism of laser action

The mechanism underlying laser-assisted debonding is primarily associated with the interaction of laser energy with bracket and adhesive materials. Although metallic brackets reflect part of the laser energy, adequate thermal conduction through the bracket base allows localized heating and softening of the adhesive interface. In contrast, ceramic brackets exhibit lower thermal conductivity and higher translucency, which may limit energy absorption and explain the minimal reduction in SBS observed in this group.

The softening of the resin matrix under laser exposure is thought to result from localized thermal expansion and partial degradation of the adhesive polymer, thereby facilitating bracket removal at lower mechanical stress levels. These observations are consistent with previous findings [17, 18].

### Enamel surface morphology

Scanning electron microscopy (SEM) analysis further supported the protective role of diode laser irradiation on enamel integrity. Control specimens exhibited distinct surface irregularities, cracks, and residual adhesive remnants, whereas laser-treated specimens showed smoother, more homogeneous enamel surfaces, indicative of reduced mechanical damage.

Comparable results have been reported in earlier studies, where laser-induced softening of adhesive resin minimized the risk of enamel microcracks and improved the ease of adhesive removal [19, 20]. These outcomes suggest that diode laser–assisted debonding not only facilitates efficient bracket removal but also contributes to superior enamel preservation and improved post-treatment aesthetics.

### Clinical implications

Clinically, the results highlight that diode laser–assisted debonding may serve as an effective adjunctive technique, particularly for metallic brackets. The observed reduction in the required debonding force may decrease the risk of enamel fractures and enhance patient comfort during bracket removal. Nevertheless, clinicians must adhere to evidence-based safety parameters to avoid excessive thermal effects within the pulp chamber. Although the present study did not measure intrapulpal temperature changes, prior ex vivo investigations have demonstrated that diode lasers operating at 1 W typically induce temperature increases below the critical threshold of 5.5 °C [14].

Accordingly, careful device calibration and operator training are essential prerequisites for the safe and effective clinical application of diode laser–assisted debonding.

### Limitations

Several limitations should be acknowledged.

First, this was an in vitro study, which does not fully replicate the complex biological conditions of the oral cavity, such as pulpal circulation, saliva, and temperature fluctuations.

Second, pulpal temperature changes were not directly measured, which limits conclusions regarding thermal safety.

Third, the sample size was relatively small, and only one power setting (1 W) and exposure duration (5 s) were evaluated.

Future studies should employ larger sample sizes, different diode laser power settings, and in vivo models to confirm the optimal parameters for safe and effective bracket debonding.

## Conclusion

Diode laser assisted debonding (Diode laser 940 nm ,1 W), is a promising tool in brackets debonding as it reduced the shear bond strength of the composite resin and reduced the occurrence of enamel cracks, thus it is a safer method for bracket debonding. But it should be used with the proper parameters, to be a safe method for preserving tooth tissue. Further research upon this field is mandatory.

## Data Availability

On reasonable request, the datasets utilized and analyzed during the present study are accessible from the corresponding author.

## Abbreviations

SBS: Shear Bond Strength and
SEM: Scanning Electron Microscopy
FIG: Figure
